# Supplementary figures and images for: Functional Cargo in Membrane Vesicles From a Citrus Pathogen
Source: Environ Microbiol Rep. 2025 Jul 22;17(4):e70101. doi: 10.1111/1758-2229.70101 (PMC12280403; doi:10.1111/1758-2229.70101)

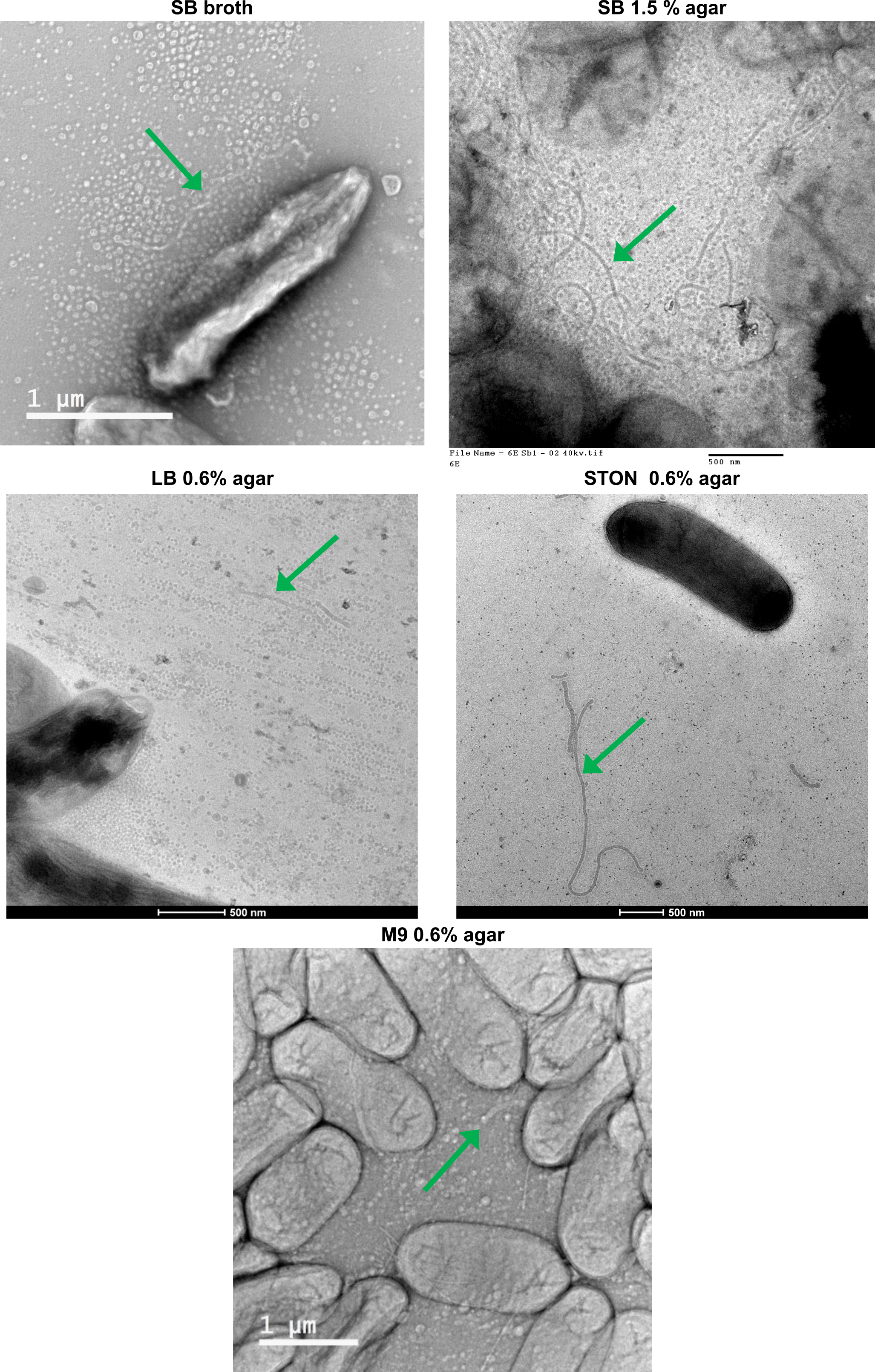

Supplement: Supplementary file 3 — Figure S1. Formation of outer membrane tubes by X. citri cells in different culture conditions and media. The tested media include liquid SB, in which the samples were concentrated by ultracentrifugation before being applied to the TEM grids, SB with 1.5% agar (a higher concentration than the 0.6% used for Figure 1), LB with 0.6% agar, STON with 0.6% agar (Guzzo et al. Journal of Molecular Biology, 2009, 10.1016/j.jmb.2009.07.065), and M9 with 0.6% agar. The green arrows point to examples of the outer membrane tubes that can be seen in the images. [file EMI4-17-e70101-s005.png]

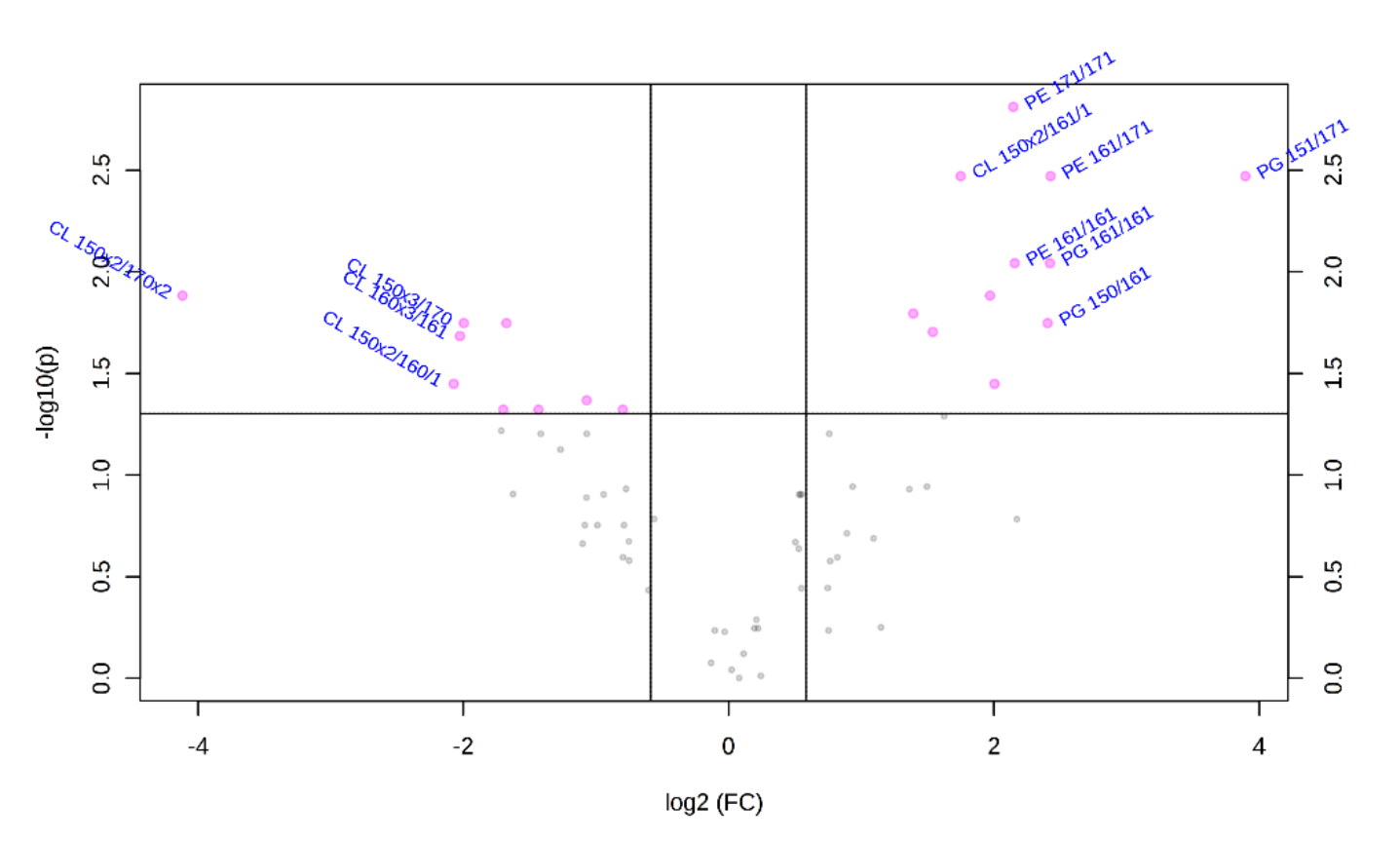

Supplement: Supplementary file 4 — Figure S2. Volcano plot analysis of the lipidomic data. The 20 most altered lipids between the OMV and whole cell samples are identified in the plot as the ones presenting fold change values above 1.5 and p < 0.05. Statistical significance was evaluated by FDR‐adjusted t‐test. [file EMI4-17-e70101-s006.png]
