# Supplementary material for: Functional Cargo in Membrane Vesicles From a Citrus Pathogen
Source: Environ Microbiol Rep. 2025 Jul 22;17(4):e70101. doi: 10.1111/1758-2229.70101 (PMC12280403; doi:10.1111/1758-2229.70101)
Supplement: Supplementary file 6 — Table S1. Combination of the top 100 most abundant proteins determined by their iBAQ values from the different purified X. citri OMV samples (gel bands and a replicate of samples in solution, Figure 4), resulting in a list of 163 non‐redundant proteins. UniProt annotations are presented for each sequence. Table S2. Results from the TQ ICP‐MS elemental analysis of samples containing purified OMV suspended in PBS. The data for Figure 7B were obtained by subtracting the background concentration of each element in PBS and normalising the values for each sample based on their respective carbon content. See also Tables S3 and S4 for experimental details. LOD: limit of detection. Table S3. Mass values defined in the quadrupoles for the TQ ICP‐MS elemental analysis. Table S4. TQ ICP‐MS operating conditions. [file EMI4-17-e70101-s003.docx]

**Table S1.** Combination of the top 100 most abundant proteins determined by their iBAQ values from the different purified *X. citri* OMV samples (gel bands and a replicate of samples in solution, **Fig. 4**), resulting in a list of 163 non-redundant proteins. UniProt annotations are presented for each sequence.

| UniProt ID | Gene names | Locus tags | Protein names (UniProt) | Pfam domains | InterPro domains |
| --- | --- | --- | --- | --- | --- |
| Q8PRF7 |  | XAC0006 | Peptidase_M48 domain-containing protein | PF01435 | IPR001915 |
| Q8PRF6 |  | XAC0007 | TPR_REGION domain-containing protein | PF13181 | IPR013026; IPR011990; IPR019734 |
| Q8PRF4 | exbB | XAC0009 | Biopolymer transport ExbB protein | PF01618 | IPR002898 |
| Q8PRE0 | ctp | XAC0023 | Carboxyl-terminal protease | PF13180; PF03572 | IPR029045; IPR001478; IPR036034; IPR004447; IPR005151 |
| Q8PRD3 | egl | XAC0030 | Cellulase | PF00150 | IPR001547; IPR018087; IPR017853 |
| Q8PRC7 |  | XAC0036 | Uncharacterized protein |  |  |
| Q8PR40 | yncD | XAC0126 | Iron transporter | PF07715; PF00593 | IPR039426; IPR012910; IPR037066; IPR000531; IPR036942 |
| Q8PQZ3 | fpvA | XAC0176 | Ferripyoverdine receptor | PF07715; PF00593 | IPR012910; IPR037066; IPR039423; IPR000531; IPR036942; IPR010105 |
| Q8PQZ2 |  | XAC0177 | PNPLA domain-containing protein | PF01734 | IPR016035; IPR002641 |
| Q8PQX9 |  | XAC0190 | Uncharacterized protein |  | IPR011990 |
| Q8PQW2 | yojM | XAC0209 | Superoxide dismutase [Cu-Zn] (EC 1.15.1.1) | PF00080 | IPR036423; IPR024134; IPR018152; IPR001424 |
| Q8PQU8 |  | XAC0223 | Uncharacterized protein |  | IPR026364; IPR023614 |
| Q8PQT9 |  | XAC0232 | Uncharacterized protein | PF13698 | IPR025294 |
| Q8PQQ0 |  | XAC0272 | Uncharacterized protein |  |  |
| Q8PQN4 |  | XAC0289 | Uncharacterized protein |  | IPR016980; IPR029063 |
| Q8NL21 | rplM | XAC0487 | 50S ribosomal protein L13 | PF00572 | IPR005822; IPR005823; IPR023563; IPR036899 |
| Q8PPZ1 | groL | XAC0542 | 60 kDa chaperonin (GroEL protein) (Protein Cpn60) | PF00118 | IPR018370; IPR001844; IPR002423; IPR027409; IPR027413; IPR027410 |
| Q8PPR2 |  | XAC0623 | Uncharacterized protein | PF04338 | IPR007433 |
| Q8PPM3 | rlpA | XAC0663 | Endolytic peptidoglycan transglycosylase RlpA (EC 4.2.2.-) | PF03330; PF05036 | IPR034718; IPR009009; IPR036908; IPR012997; IPR007730; IPR036680 |
| Q8PPM2 | dacC | XAC0664 | Serine-type D-Ala-D-Ala carboxypeptidase (EC 3.4.16.4) | PF07943; PF00768 | IPR012338; IPR015956; IPR018044; IPR012907; IPR037167; IPR001967 |
| Q8PPK9 |  | XAC0677 | Uncharacterized protein | PF10001 | IPR018718 |
| Q8PPK4 |  | XAC0682 | BON domain-containing protein | PF04972 | IPR007055; IPR014004 |
| Q8PPJ6 | fecA | XAC0690 | TonB-dependent receptor | PF07715; PF00593 | IPR012910; IPR037066; IPR039423; IPR000531; IPR036942 |
| Q8PPH0 | fyuA | XAC0716 | TonB-dependent receptor | PF07715; PF00593 | IPR012910; IPR037066; IPR000531; IPR036942; IPR010104 |
| Q8PPD9 |  | XAC0747 | Uncharacterized protein |  | IPR023614 |
| Q8PPC1 |  | XAC0765 | Uncharacterized protein | PF04348 | IPR007443; IPR028082 |
| Q8PP23 | surA | XAC0865 | Chaperone SurA (Peptidyl-prolyl cis-trans isomerase SurA) (PPIase SurA) (EC 5.2.1.8) (Rotamase SurA) | PF00639; PF09312 | IPR000297; IPR023034; IPR015391; IPR027304 |
| Q8PP00 | gfo | XAC0888 | Glucose-fructose oxidoreductase | PF01408; PF02894 | IPR004104; IPR008354; IPR036291; IPR000683 |
| Q8PNT4 | rplK | XAC0961 | 50S ribosomal protein L11 | PF00298; PF03946 | IPR000911; IPR036796; IPR006519; IPR020783; IPR036769; IPR020785; IPR020784 |
| Q8PNS2 | rplW | XAC0974 | 50S ribosomal protein L23 | PF00276 | IPR012677; IPR012678; IPR013025 |
| Q8PNS1 | rplB | XAC0975 | 50S ribosomal protein L2 | PF00181; PF03947 | IPR012340; IPR022666; IPR014722; IPR002171; IPR005880; IPR022669; IPR022671; IPR014726; IPR008991 |
| Q8NKY0 | rplV | XAC0977 | 50S ribosomal protein L22 | PF00237 | IPR001063; IPR018260; IPR036394; IPR005727 |
| Q8PNR8 | rplP | XAC0979 | 50S ribosomal protein L16 | PF00252 | IPR016180; IPR036920; IPR000114; IPR020798 |
| Q8NL02 | rplN | XAC0982 | 50S ribosomal protein L14 | PF00238 | IPR036853; IPR000218; IPR005745; IPR019972 |
| Q8PNR4 | rplE | XAC0984 | 50S ribosomal protein L5 | PF00281; PF00673 | IPR002132; IPR020930; IPR031309; IPR020929; IPR022803; IPR031310 |
| Q8PNR3 | rpsH | XAC0986 | 30S ribosomal protein S8 | PF00410 | IPR000630; IPR035987 |
| Q8PNR1 | rplR | XAC0988 | 50S ribosomal protein L18 | PF00861 | IPR005484; IPR004389 |
| Q8PNR0 | rpmD | XAC0990 | 50S ribosomal protein L30 | PF00327 | IPR036919; IPR005996; IPR016082 |
| Q8PNQ9 | rplO | XAC0991 | 50S ribosomal protein L15 | PF00828 | IPR036227; IPR030878; IPR005749; IPR001196; IPR021131 |
| Q8NKX3 | rpsM | XAC0993 | 30S ribosomal protein S13 | PF00416 | IPR027437; IPR001892; IPR010979; IPR019980; IPR018269 |
| P0A0Y0 | rpsD | XAC0995 | 30S ribosomal protein S4 | PF00163; PF01479 | IPR022801; IPR001912; IPR005709; IPR018079; IPR002942; IPR036986 |
| Q8PNQ7 | rplQ | XAC0997 | 50S ribosomal protein L17 | PF01196 | IPR000456; IPR036373 |
| Q8PNP2 | mopB | XAC1012 | Outer membrane protein | PF13505; PF00691 | IPR011250; IPR027385; IPR006664; IPR006665; IPR036737; IPR028974 |
| Q8PNJ7 |  | XAC1062 | Uncharacterized protein |  |  |
| Q8PNF8 | slp | XAC1113 | Outer membrane protein Slp | PF03843 | IPR004658 |
| Q8PND0 | fyuA | XAC1143 | TonB-dependent receptor | PF07715; PF00593 | IPR012910; IPR039423; IPR000531; IPR036942 |
| Q8PNB0 |  | XAC1163 | Uncharacterized protein |  |  |
| Q8PN49 | minE | XAC1224 | Cell division topological specificity factor | PF03776 | IPR005527; IPR036707 |
| Q8PN43 |  | XAC1230 | Uncharacterized protein |  | IPR011256 |
| Q8PN35 |  | XAC1238 | Endo/exonuclease/phosphatase domain-containing protein | PF03372 | IPR036691; IPR005135 |
| Q8PN33 |  | XAC1240 | Uncharacterized protein | PF13202; PF13499 | IPR011992; IPR018247; IPR002048 |
| Q8PN25 | rplU | XAC1248 | 50S ribosomal protein L21 | PF00829 | IPR036164; IPR028909; IPR001787; IPR018258 |
| Q8PMV4 | mucD | XAC1321 | Periplasmic serine endoprotease DegP-like (EC 3.4.21.107) | PF13180 | IPR001478; IPR036034; IPR011782; IPR009003; IPR001940 |
| Q8PMV1 |  | XAC1324 | Uncharacterized protein | PF16137 | IPR032314 |
| Q8PMS7 |  | XAC1349 | Serine protease | PF03797; PF12951; PF00082 | IPR005546; IPR036709; IPR013425; IPR000209; IPR036852; IPR023827; IPR023828; IPR015500; IPR034061 |
| Q8PMP7 |  | XAC1379 | Uncharacterized protein | PF10099 | IPR018764 |
| Q8PMM9 |  | XAC1397 | Alginate_exp domain-containing protein | PF13372 | IPR025388 |
| Q8PML3 | oma | XAC1413 | Outer membrane protein assembly factor BamA | PF01103; PF07244 | IPR000184; IPR010827; IPR039910; IPR023707; IPR034746 |
| Q8PMJ3 |  | XAC1434 | CASH domain-containing protein | PF13229 | IPR039448; IPR006633; IPR022441; IPR006626; IPR012334; IPR011050 |
| Q8PMJ2 | fhuA | XAC1435 | Iron receptor | PF07715; PF00593 | IPR012910; IPR037066; IPR039423; IPR000531; IPR036942; IPR010917; IPR010105 |
| Q8PMH2 | dcp | XAC1456 | Peptidyl-dipeptidase | PF01432 | IPR034005; IPR024077; IPR001567 |
| Q8PMG5 |  | XAC1463 | Phospholipase A1 (EC 3.1.1.32) (EC 3.1.1.4) (Phosphatidylcholine 1-acylhydrolase) | PF02253 | IPR003187; IPR036541 |
| Q8PMG3 | pcp | XAC1466 | Peptidoglycan-associated outer membrane lipoprotein | PF05433 | IPR008816 |
| Q8PMF0 |  | XAC1479 | OmpA family protein | PF13488; PF00691 | IPR039567; IPR006664; IPR006665; IPR006690; IPR036737 |
| Q8PMD2 |  | XAC1497 | Uncharacterized protein |  |  |
| Q8PMB6 | smpA | XAC1516 | Outer membrane protein assembly factor BamE | PF04355 | IPR026592; IPR037873; IPR007450 |
| Q8PM83 | btuE | XAC1549 | Glutathione peroxidase | PF00255 | IPR000889; IPR029759; IPR036249 |
| Q8PM82 | fkpA | XAC1550 | Peptidyl-prolyl cis-trans isomerase (EC 5.2.1.8) | PF00254; PF01346 | IPR001179; IPR000774; IPR036944 |
| Q8PM54 | oprO | XAC1579 | Polyphosphate-selective porin O | PF07396 | IPR023614; IPR010870 |
| Q8NL26 |  | XAC1585 | Peptidyl-prolyl cis-trans isomerase (EC 5.2.1.8) | PF00254; PF01346 | IPR001179; IPR000774; IPR036944 |
| Q8PM13 | rpsR | XAC1621 | 30S ribosomal protein S18 | PF01084 | IPR001648; IPR018275; IPR036870 |
| Q8PLS7 |  | XAC1712 | DUF218 domain-containing protein | PF02698 | IPR003848; IPR014729 |
| Q8PLR1 | nlpD | XAC1728 | Lipoprotein | PF01476; PF01551 | IPR011055; IPR018392; IPR036779; IPR016047 |
| Q8PLN4 |  | XAC1761 | Uncharacterized protein |  |  |
| Q8PL93 | cirA | XAC1910 | TonB-dependent receptor | PF07715; PF00593 | IPR012910; IPR037066; IPR000531; IPR010104 |
| Q8PKZ8 | lolA | XAC2008 | Outer-membrane lipoprotein carrier protein | PF03548 | IPR029046; IPR004564; IPR018323 |
| Q8PKZ0 | pilF | XAC2017 | Fimbrial biogenesis protein |  | IPR013360; IPR013026; IPR011990; IPR019734 |
| Q8PKY7 | bamB | XAC2020 | Outer membrane protein assembly factor BamB | PF13360 | IPR017687; IPR018391; IPR002372; IPR011047; IPR015943 |
| Q8PK64 |  | XAC2312 | TonB_dep_Rec domain-containing protein | PF00593 | IPR039426; IPR013784; IPR000531 |
| Q8PK57 |  | XAC2319 | Uncharacterized protein |  |  |
| Q8PK24 |  | XAC2353 | Uncharacterized protein |  |  |
| Q8PJM6 | rpfN | XAC2504 | Porin | PF04966 | IPR007049; IPR038673 |
| Q8PJK8 | ggt | XAC2523 | Gamma-glutamyltranspeptidase |  | IPR043138; IPR000101; IPR043137; IPR029055 |
| Q8PJK6 |  | XAC2525 | Uncharacterized protein |  |  |
| Q8PJK0 | btuB | XAC2531 | TonB-dependent receptor | PF07715; PF00593 | IPR012910; IPR037066; IPR000531; IPR010916 |
| Q8PJH0 |  | XAC2562 | Uncharacterized protein |  |  |
| Q8PJE3 | rplT | XAC2591 | 50S ribosomal protein L20 | PF00453 | IPR005813; IPR035566 |
| Q8PJD5 | btuB | XAC2600 | TonB-dependent receptor | PF07715 | IPR012910; IPR037066; IPR010104; IPR010917 |
| Q8PJC4 |  | XAC2611 | DUF4189 domain-containing protein | PF13827 | IPR025240 |
| Q8PJB5 | virB9 | XAC2620 | VirB9 protein | PF03524 | IPR010258; IPR033645; IPR038161 |
| Q8PJ70 | oar | XAC2672 | Oar protein |  | IPR039426; IPR008969; IPR036942 |
| Q8PJ58 | rpsO | XAC2684 | 30S ribosomal protein S15 | PF00312 | IPR000589; IPR005290; IPR009068 |
| Q8PJ03 | btuB | XAC2742 | TonB-dependent receptor | PF07715; PF00593 | IPR012910; IPR037066; IPR000531; IPR010917 |
| Q8PJ02 | oar | XAC2743 | Oar protein | PF07715; PF00593 | IPR039426; IPR013784; IPR012910; IPR037066; IPR000531 |
| Q8PIY6 | phoA | XAC2759 | Alkaline phosphatase | PF00245 | IPR001952; IPR017850 |
| Q8PIX3 | bp26 | XAC2772 | Outer membrane protein | PF04402 | IPR007497 |
| Q8PIX2 | oar | XAC2773 | Oar protein | PF07715; PF00593 | IPR039426; IPR013784; IPR012910; IPR037066; IPR000531 |
| Q8PIW5 | rlpB | XAC2780 | LPS-assembly lipoprotein LptE | PF04390 | IPR007485 |
| Q8PIU4 |  | XAC2801 | Uncharacterized protein | PF06629 | IPR010583 |
| Q8PIR6 | phuR | XAC2829 | Outer membrane hemin receptor | PF07715; PF00593 | IPR039426; IPR012910; IPR037066; IPR000531; IPR036942 |
| Q8PIF7 | fhuA | XAC2941 | TonB-dependent receptor | PF07715; PF00593 | IPR012910; IPR037066; IPR039423; IPR000531; IPR036942; IPR010105 |
| Q8PIF2 |  | XAC2946 | Uncharacterized protein | PF10670 | IPR019613 |
| Q8PIE7 | comEA | XAC2951 | DNA transport competence protein |  | IPR004509; IPR010994 |
| Q8PIE0 |  | XAC2958 | Uncharacterized protein | PF09839 | IPR018642 |
| Q8PID5 |  | XAC2963 | Uncharacterized protein | PF11306 | IPR021457 |
| Q8PI48 | btuB | XAC3050 | TonB-dependent receptor | PF07715; PF00593 | IPR012910; IPR037066; IPR000531; IPR036942 |
| Q8PI41 | bla | XAC3057 | Beta-lactamase | PF00144 | IPR001466; IPR012338 |
| Q8PI27 | iroN | XAC3071 | TonB-dependent receptor | PF07715; PF00593 | IPR012910; IPR037066; IPR000531; IPR010104 |
| Q8PHZ0 |  | XAC3108 | Uncharacterized protein |  | IPR011990 |
| Q8PHV9 | cpoB | XAC3140 | Cell division coordinator CpoB | PF16331; PF13525 | IPR039565; IPR034706; IPR014162; IPR013026; IPR011990; IPR019734; IPR032519 |
| Q8PHV8 | ompP6 | XAC3141 | Peptidoglycan-associated protein | PF00691 | IPR006664; IPR006665; IPR036737; IPR039001; IPR014169 |
| Q8PHV7 | tolB | XAC3142 | Tol-Pal system protein TolB | PF07676; PF04052 | IPR011042; IPR011659; IPR014167; IPR007195; IPR036752 |
| Q8PHV5 | tolR | XAC3144 | Tol-Pal system protein TolR | PF02472 | IPR003400; IPR014168 |
| Q8PHV4 | tolQ | XAC3145 | Tol-Pal system protein TolQ | PF01618 | IPR002898; IPR014163 |
| Q8PHU4 |  | XAC3155 | Uncharacterized protein | PF11218 | IPR021381 |
| Q8PHT7 | bla | XAC3162 | Beta-lactamase (EC 3.5.2.6) |  | IPR012338; IPR000871; IPR023650; IPR006311 |
| Q8PHT1 | bfeA | XAC3168 | Ferric enterobactin receptor | PF07715; PF00593 | IPR012910; IPR037066; IPR000531; IPR010916; IPR036942 |
| Q8PHT0 | bfeA | XAC3169 | Ferric enterobactin receptor | PF07715; PF00593 | IPR012910; IPR037066; IPR000531; IPR036942 |
| Q8PHS3 | fecA | XAC3176 | Citrate-dependent iron transporter | PF07715; PF00593 | IPR012910; IPR037066; IPR000531; IPR036942; IPR010105 |
| Q8PHQ5 | btuB | XAC3194 | Outer membrane receptor for transport of vitamin B | PF07715; PF00593 | IPR010101; IPR039426; IPR012910; IPR037066; IPR000531; IPR036942 |
| Q8PHP1 | bfeA | XAC3207 | Ferric enterobactin receptor | PF07715; PF00593 | IPR012910; IPR037066; IPR000531; IPR010916; IPR036942 |
| Q8PHN1 | comL | XAC3218 | Outer membrane protein assembly factor BamD | PF13525 | IPR017689; IPR039565; IPR013026; IPR011990 |
| Q8PHL0 | fimA | XAC3241 | Fimbrillin | PF07963; PF00114 | IPR012902; IPR001082 |
| Q8PHF7 | estA | XAC3300 | Lipase | PF03797; PF00657 | IPR005546; IPR036709; IPR001087; IPR017186; IPR036514 |
| Q8PHE6 | iroN | XAC3311 | TonB-dependent receptor | PF07715; PF00593 | IPR012910; IPR037066; IPR006311; IPR000531; IPR036942; IPR010104 |
| Q8PHC5 | fecA | XAC3334 | TonB-dependent receptor | PF07715; PF00593 | IPR039426; IPR012910; IPR037066; IPR000531; IPR036942 |
| Q8PHA8 |  | XAC3351 | Uncharacterized protein |  |  |
| Q8PHA5 | ompW | XAC3354 | Outer membrane protein W | PF03922 | IPR011250; IPR005618 |
| Q8PHA4 | omp21 | XAC3355 | Outer membrane protein | PF03922 | IPR011250; IPR005618 |
| Q8PH89 | fhuE | XAC3370 | Outer membrane receptor for ferric iron uptake | PF07715; PF00593 | IPR012910; IPR037066; IPR039423; IPR000531; IPR036942; IPR010105 |
| Q8PH16 | btuB | XAC3444 | TonB-dependent receptor | PF07715; PF00593 | IPR012910; IPR037066; IPR000531; IPR036942 |
| Q8PGZ9 | tolC | XAC3463 | TolC protein | PF02321 | IPR003423; IPR010130 |
| Q8PGZ0 | oprO | XAC3472 | Polyphosphate-selective porin O | PF07396 | IPR023614; IPR010870 |
| Q8PGX3 | fyuA | XAC3489 | TonB-dependent receptor | PF07715; PF00593 | IPR012910; IPR037066; IPR039423; IPR000531; IPR036942 |
| Q8PGW4 | fhuE | XAC3498 | Outer membrane receptor for ferric iron uptake | PF07715; PF00593 | IPR012910; IPR037066; IPR039423; IPR000531; IPR036942; IPR010917; IPR010105 |
| Q8PGU1 |  | XAC3525 | Uncharacterized protein |  |  |
| Q8PGL1 | uptE | XAC3605 | Outer membrane protein |  | IPR036737 |
| Q8PGL0 | uptD | XAC3606 | Outer membran protein | PF14346 | IPR025511 |
| Q8PGJ6 | pfeA | XAC3620 | Siderophore receptor protein | PF07715; PF00593 | IPR039426; IPR012910; IPR037066; IPR000531; IPR036942; IPR010917; IPR010105 |
| Q8PGG6 | atpG | XAC3650 | ATP synthase gamma chain (ATP synthase F1 sector gamma subunit) | PF00231 | IPR035968; IPR000131; IPR023632 |
| Q8PGG0 |  | XAC3657 | Uncharacterized protein |  | IPR011250 |
| Q8PGF7 |  | XAC3660 | Uncharacterized protein |  |  |
| Q8PGF3 | ompW | XAC3664 | Outer membrane protein | PF03922 | IPR011250; IPR005618 |
| Q8PGF0 |  | XAC3667 | Lipoprotein | PF03180 | IPR004872 |
| Q8PGC9 | dadA | XAC3688 | D-amino acid dehydrogenase (EC 1.4.99.-) | PF01266 | IPR023080; IPR006076; IPR036188 |
| Q8PFX5 | amaA | XAC3847 | N-acyl-L-amino acid amidohydrolase | PF07687; PF01546 | IPR017439; IPR036264; IPR002933; IPR011650 |
| Q8PFW2 |  | XAC3860 | N-acetylmuramoyl-L-alanine amidase | PF01510 | IPR036505; IPR002502 |
| Q8PFV4 | yliI | XAC3868 | Dehydrogenase | PF07995 | IPR011042; IPR012938; IPR011041 |
| P66535 | rpsU | XAC3872 | 30S ribosomal protein S21 | PF01165 | IPR001911; IPR018278; IPR038380 |
| Q8PFR1 |  | XAC3917 | SPOR domain-containing protein | PF05036 | IPR007730; IPR036680 |
| Q8PFQ2 |  | XAC3926 | OMP_b-brl domain-containing protein | PF13505 | IPR011250; IPR027385 |
| Q8PFK1 | htrA | XAC3980 | Periplasmic serine endoprotease DegP-like (EC 3.4.21.107) | PF13180 | IPR001478; IPR036034; IPR011782; IPR009003; IPR001940 |
| Q8PFK0 |  | XAC3981 | Uncharacterized protein |  |  |
| Q8PFH3 | ecnA | XAC4008 | Entericidin A | PF08085 | IPR012556 |
| Q8PFD5 | iroN | XAC4048 | TonB-dependent receptor | PF07715; PF00593 | IPR012910; IPR037066; IPR000531; IPR010104 |
| P66160 | rpmB | XAC4159 | 50S ribosomal protein L28 | PF00830 | IPR034704; IPR026569; IPR037147; IPR001383 |
| Q8PEX1 |  | XAC4219 | Ysc84 domain-containing protein | PF04366 | IPR007461 |
| Q8PER7 |  | XAC4273 | OmpA-related protein | PF00593 | IPR039426; IPR013784; IPR000531 |
| Q8PER6 |  | XAC4274 | OmpA-related protein | PF00593 | IPR039426; IPR013784; IPR000531 |
| Q8PEK7 | yrbC | XAC4342 | Toluene tolerance protein | PF05494 | IPR008869; IPR042245 |
| Q8PEK5 | vacJ | XAC4344 | Lipoprotein | PF04333 | IPR007428 |
| Q8PRJ3 | virB9 | XACb0039 | VirB9 protein | PF03524 | IPR010258; IPR014148; IPR033645; IPR038161 |
| Q8NL05 | rpsN |  | 30S ribosomal protein S14 | PF00253 | IPR001209; IPR043140; IPR023036 |

**Table S2.** Results from the TQ ICP-MS elemental analysis of samples containing purified OMV suspended in PBS. The data for **Fig. 7B** were obtained by subtracting the background concentration of each element in PBS and normalizing the values for each sample based on their respective carbon content. See also **Table S3** and **Table S4** for experimental details. LOD: limit of detection.

| **Element** | **Buffer PBS 1×** | | | **Sample 1** | | | **Sample 2** | | | **Sample 3** | | | **LOD** | **Unit** |
| --- | --- | --- | --- | --- | --- | --- | --- | --- | --- | --- | --- | --- | --- | --- |
| C | < LOD | | | 776 | ± | 13 | 1285 | ± | 3 | 899 | ± | 2 | 24.8 | **mg L^-1^** |
| Mg | < LOD | | | 41.4 | ± | 0.6 | 80.6 | ± | 1.0 | 51.2 | ± | 1.0 | 0.295 | µg L^-1^ |
| S | 80 | ± | 1 | 112 | ± | 2 | 152 | ± | 3 | 113 | ± | 2 | 0.473 | µg L^-1^ |
| Ca | 47 | ± | 1 | 273 | ± | 3 | 292 | ± | 10 | 232 | ± | 6 | 6.782 | µg L^-1^ |
| Mn | 0.09 | ± | 0.01 | 0.43 | ± | 0.01 | 0.62 | ± | 0.03 | 0.54 | ± | 0.01 | 0.007 | µg L^-1^ |
| Fe | 1.58 | ± | 0.14 | 2.13 | ± | 0.03 | 2.32 | ± | 0.10 | 2.85 | ± | 0.14 | 0.196 | µg L^-1^ |
| Co | 0.044 | ± | 0.003 | 0.053 | ± | 0.005 | 0.052 | ± | 0.003 | 0.059 | ± | 0.006 | 0.001 | µg L^-1^ |
| Ni | 0.48 | ± | 0.02 | 3.08 | ± | 0.09 | 0.96 | ± | 0.06 | 0.97 | ± | 0.10 | 0.154 | µg L^-1^ |
| Cu | < LOD | | | 0.132 | ± | 0.002 | < LOD | | | < LOD | | | 0.037 | µg L^-1^ |
| Zn | 1.06 | ± | 0.03 | 6.97 | ± | 0.10 | 8.04 | ± | 0.15 | 14.57 | ± | 0.12 | 0.175 | µg L^-1^ |
| Br | 153 | ± | 8 | 136 | ± | 14 | 149 | ± | 11 | 138 | ± | 7 | 0.042 | µg L^-1^ |
| Se | < LOD | | | < LOD | | | 0.004 | ± | 0.001 | < LOD | | | 0.002 | µg L^-1^ |
| Ba | 1.01 | ± | 0.03 | 1.15 | ± | 0.01 | 1.49 | ± | 0.02 | 1.27 | ± | 0.02 | 0.003 | µg L^-1^ |

**Table S3.** Mass values defined in the quadrupoles for the TQ ICP-MS elemental analysis.

| **Element** | **Isotope** | **Analysis Mode** | **Q_1_ mass** | **Q_2_ filled with** | **Q_3_ mass** | **LOD** | **Unit** | **Linear Range** | **Sensitivity (cps L µg^-1^)** | **R^2^** |
| --- | --- | --- | --- | --- | --- | --- | --- | --- | --- | --- |
| C | 12 | SQ - KED | --- | Helium | 12 | 24.8 | **mg L^-1^** | 50 - 1500 | 3.0 × 10^2^ | 0.9999 |
| Mg | 24 | SQ - KED | --- | Helium | 24 | 0.295 | µg L^-1^ | 25 - 100 | 6.3 × 10^2^ | 0.9924 |
| S | 32 | TQ - O2 | 32 | Oxygen | 48 (^32^S.^16^O^+^) | 0.473 | µg L^-1^ | 3 - 160 | 1.9 × 10^3^ | 0.9987 |
| Ca | 44 | SQ - KED | --- | Helium | 44 | 6.782 | µg L^-1^ | 25 - 500 | 4.5 × 10^1^ | 0.9997 |
| Mn | 55 | SQ - KED | --- | Helium | 55 | 0.007 | µg L^-1^ | 0.01 - 5 | 9.2 × 10^3^ | 0.9999 |
| Fe | 57 | SQ - KED | --- | Helium | 57 | 0.196 | µg L^-1^ | 0.25 - 5 | 4.1 × 10^2^ | 0.9977 |
| Co | 59 | SQ - KED | --- | Helium | 59 | 0.001 | µg L^-1^ | 0.01 - 1 | 3.8 × 10^4^ | 0.9993 |
| Ni | 60 | SQ - KED | --- | Helium | 60 | 0.154 | µg L^-1^ | 0.25 - 10 | 1.2 × 10^4^ | 0.9939 |
| Cu | 63 | SQ - KED | --- | Helium | 63 | 0.037 | µg L^-1^ | 0.05 - 10 | 3.1 × 10^4^ | 0.9999 |
| Zn | 66 | SQ - KED | --- | Helium | 66 | 0.175 | µg L^-1^ | 0.25 - 25 | 4.1 × 10^3^ | 0.9997 |
| Br | 79 | TQ - O2 | 79 | Oxygen | 95 (^79^Br.^16^O^+^) | 0.042 | µg L^-1^ | 50 - 500 | 3.6 × 10^2^ | 0.9999 |
| Se | 80 | TQ - O2 | 80 | Oxygen | 96 (^80^Se.^16^O^+^) | 0.002 | µg L^-1^ | 0.005 - 1 | 2.1 × 10^3^ | 0.9993 |
| Ba | 138 | TQ - O2 | 138 | Oxygen | 154 (^138^Ba.^16^O^+^) | 0.003 | µg L^-1^ | 0.05 - 5 | 5.2 × 10^4^ | 0.9999 |

**Table S4.** TQ ICP-MS operating conditions.

| RF Power (W) | 1550 |
| --- | --- |
| Argon coolant gas flow (L min^-1^) | 14 |
| Argon auxiliary gas flow (L min^-1^) | 0.8 |
| Argon nebulizer flow (L min^-1^) | 1.04 |
| He flow gas (mL min^-1^) | 6.57 |
| O_2_ flow gas (mL min^-1^) | 0.6 |
| Nebulizer | MicroMist U-Series 0.4 mL min^-1^ |
| Spray Chamber | Glass Cyclonic spray chamber |
| Peristaltic Pump (rpm) | 40 |
| Spray chamber temperature ($\boldsymbol{℃}$) | 2.7 |
| Dwell time (s) | 0.1 |
| Number of Sweeps | 10 |

**Fig. S1.** Formation of outer membrane tubes by *X. citri* cells in different culture conditions and media. The tested media include liquid SB, in which the samples were concentrated by ultracentrifugation before being applied to the TEM grids, SB with 1.5% agar (a higher concentration than the 0.6% used for Fig. 1), LB with 0.6% agar, STON with 0.6% agar (Guzzo et al., J Mol Biol, 2009, 10.1016/j.jmb.2009.07.065), and M9 with 0.6% agar. The green arrows point to examples of the outer membrane tubes that can be seen in the images.

**Fig. S2.** Volcano plot analysis of the lipidomic data. The 20 most altered lipids between the OMV and whole cell samples are identified in the plot as the ones presenting fold change values above 1.5 and p<0.05. Statistical significance was evaluated by FDR-adjusted t-test.

**Fig. S3.** Most significantly enriched InterPro domains found in the purified OMVs compared to the *X. citri* pv. *citri* 306 genome. The lowest false discovery rates (FDR), thus the highest -log10(FDR) values, were observed for domains related to TonB-dependent receptors.

**Data Set S1.** Proteomic data for purified *X. citri* OMVs, containing details of the filtered proteins identified for the duplicate of in-solution digestions, including their iBAQ values (XLSX file).

**Data Set S2.** Proteomic data for purified *X. citri* OMVs, containing details of the filtered proteins identified for the in-gel digestion, including their iBAQ values (XLSX file).
